# Impact of musculoskeletal disorders on healthy life expectancy in Japan

**DOI:** 10.1186/s12891-021-04539-4

**Published:** 2021-08-06

**Authors:** Yoshihiro Ritsuno, Miyuki Kawado, Mitsuhiro Morita, Harumoto Yamada, Arihiko Kanaji, Masaya Nakamura, Morio Matsumoto, Shuji Hashimoto, Nobuyuki Fujita

**Affiliations:** 1 Department of Orthopaedic Surgery, Fujita Health University School of Medicine, 1-98 Dengakugakubo, Kutsukake-cho, Toyoake, Aichi 470-1192 Japan; 2 Department of Orthopaedic Surgery, Keio University School of Medicine, Tokyo, Japan; 3 Department of Hygiene, Fujita Health University School of Medicine, Aichi, Japan

**Keywords:** Disability-free life expectancy, Healthy life expectancy, Life expectancy, Activities of daily living, Health statistics

## Abstract

**Background:**

Musculoskeletal disorders are a key cause of morbidity in elderly people. How musculoskeletal disorders relate to healthy life expectancy remain elusive. Hence, we aimed to estimate gains in healthy life expectancy from the elimination of musculoskeletal diseases and injuries by using recent national health statistics data in Japan.

**Methods:**

Mortality data were taken from Japanese national life tables and death certificates in 2016. Information on medical diagnoses, injuries, and activity were obtained from the 2016 Comprehensive Survey of Living Conditions. We examined five disorders: rheumatoid arthritis, arthrosis, low back pain, osteoporosis, and fracture. The prevalence of limitations in activities of daily living (ADL) in the population after eliminating the disorder was estimated as the proportion of outpatients without the disorder and ADL limitations, inpatients without the disorder in hospitals and clinics, and people without the disorder who reside in long-term elderly care facilities.

**Results:**

There were small gains in life expectancy from elimination of all selected musculoskeletal disorders (0.0–0.1 years). Elimination of rheumatoid arthritis, osteoporosis, and fracture slightly increased the expected years without activity limitation (0.1–0.4) and slightly decreased years with activity limitation (0.1–0.4 years). Meanwhile, elimination of arthrosis, low back pain, and arthrosis and low back pain moderately increased expected years without activity limitation (0.3–1.5 years) and decreased years with activity limitation (0.3–1.5 years). In addition, elimination of rheumatoid arthritis, arthrosis, low back pain, osteoporosis, and fracture decreased expected years with ADL limitations (0.0–0.8 years) and non-ADL limitations (0.0–0.3 years). A combination of arthrosis and low back pain showed a moderate decrease in expected years with both ADL limitations (0.7–1.1 years) and non-ADL limitations (0.3–0.4).

**Conclusions:**

These findings provide clinical evidence that among the musculoskeletal disorders low back pain and arthrosis are the key factors for the elongation of healthy life expectancy.

## Introduction

The world is seeing a rapid increase in the aging population compared to years past. Worldwide, 22% (2 billion) of the population is expected to be over 60 years of age by 2050 up from 12% (900 million) in 2015. Changes in the proportion of the population older than 60 years have been observed in many countries in the past few decades [1]. The World Health Organization (WHO) has estimated that 40% of people over 60 years of age have experienced musculoskeletal disorders and 80% have had low back pain at some point in their lives [1, 2]. Musculoskeletal disorders are a key cause of morbidity in elderly people [3]. The musculoskeletal system regulates the ability to move, and overcoming musculoskeletal disorders may extend healthy life expectancy.

Healthy life expectancy is an intuitive and meaningful measure of population health and represents a long and healthy life lived [4, 5]. Among several indices for healthy life expectancy [6], the most popular index is life expectancy without activity limitation, calculated by the Sullivan method [7–9]. Japan was the top country with the world’s longest life expectancy for both sexes in 2013, and both life expectancy and healthy life expectancy had increased significantly since 1990 [10]. Even in the country, however, men and women are expected to spend roughly a decade of their lives – typically in older age – with at least some activity limitations [11]. This gap between life expectancy and healthy life expectancy creates a considerable economic and social burden [1].

The previous reports [12, 13] implicated musculoskeletal disorders as adverse factors for healthy life expectancy. However, few studies have formally estimated the association between musculoskeletal diseases and injuries and healthy life expectancy [14, 15]. One approach to examining the potential impacts at the population level is to estimate healthy life expectancy under an idealized counterfactual scenario in which musculoskeletal disease and injuries have been completely eliminated. The difference between this counterfactual and the empirical healthy life expectancy has been proposed as an indicator of disease burden [16, 17]. We have previously reported on such counterfactual scenarios eliminating a broad selection of diseases and injuries [14]. This study aims to estimate gains in healthy life expectancy by eliminating specific musculoskeletal diseases and injuries using the recent national health statistics data in Japan.

## Methods

### Data

Mortality data were obtained from Japanese national life tables and death certificates in 2016 [18, 19]. Data relating to disease and activity status of persons living at home were obtained from the 2016 Comprehensive Survey of Living Conditions [11]. These data were collected using self-administered questionnaires distributed to 710,000 people in randomly selected households nationwide. Data for persons admitted to hospitals and clinics were obtained from the Patient Surveys in 2014 and 2017, which included information on 5,000,000 randomly selected patients throughout Japan [20]. Data of 110,000 elderly individuals admitted to healthcare and welfare facilities for long-term care (“residents of long-term elder care facilities” hereafter) were obtained from the 2016 Survey of Institutions and Establishments for Long-term Care [21]. Data from all three surveys were used with the approval of the Ministry of Health, Labour and Welfare of Japan, and the Ministry of Internal Affairs and Communications.

### Activity limitation

For persons living at home, activity status was determined based on their replies to the survey questions: “Is your current daily life affected by health problems?” and “How is it affected?” [11]. Participants who responded “yes” proceeded to the second question. There were various responses to the second question, including limitations in “activities of daily living (ADL) (rising, dressing/undressing, eating, bathing, etc.),” “going out,” “work, housework, or schoolwork,” “physical exercise (including sports),” and “other.” Responses were classified into three levels of activity: (i) ADL limitation, (ii) non-ADL limitation, and (iii) no activity limitation. All persons admitted to hospitals and clinics as inpatients and residents of long-term elderly care facilities were considered to have ADL limitations.

## Disease status

We selected five disorders: rheumatoid arthritis (International Classification of Diseases, 10th Revision [ICD-10] code: M05–M06), arthrosis (M15-M19), low back pain (M40–43, M45–49, M50–51, M53.0, M54.3-M54.5), osteoporosis (M80-M82), and fracture (S02, S12, S22, S32, S42, S52, S62, S72, S82, S92, T02, T08, T10, T12, T14.2). For persons living at home, disease status was assessed using responses to the following questions: “Are you currently seeking care at a hospital, clinic, or facility of traditional Japanese massage, acupuncture, moxibustion, or judo-orthopedics for diseases or injuries?” and “What are your diseases or injuries?” [11]. Persons who replied “Yes” proceeded to the next question, which has three options: “any of the 5 disorders (39 diseases and injuries categorized under the 5 disorders),” “other disorders,” and “unknown.” Persons who responded to have any of the five disorders were classified as outpatients. For persons who are in hospitals and clinics as inpatients and residents of long-term elderly care facilities, their primary medical condition was used to determine the presence or absence of the five disorders [20, 21].

### Calculation of gains in years with and without activity limitation expected from elimination of disorders

Anticipated years of life with and without activity limitation that would be gained from eliminating each of the above five disorders in Japan as of 2016 were calculated. As with our previous report [14], gains were calculated as the difference in the number of years with versus without the disease, based on a previous study by Colvez et al. [16].

By using the data regarding the number of deaths and life tables without disease elimination, we constructed a life table that eliminated deaths caused by disease. We expressed the counterfactual probability of survival in age group *x* with the disease eliminated (*p*_*x*_^*e*^) based on the actual probability of survival without disease elimination (*p*_*x*_), the number of deaths (*D*_*x*_) from all diseases and injuries, and the number of deaths from the disease (*D*_*x*_^*e*^), as follows:

$$ \ln \left({p}_x^e\right)=\left(1-{D}_x^e/{D}_x\right)\ln \left({p}_x\right) $$

Here, ln is a natural logarithm function, and the age groups are 0 to 4, 5 to 9,…, 80 to 84, and 85 years or older. According to Chiang’s life table method [22], we calculated the number of survivors (*l*_*x*_^*e*^) and the stationary population (*L*_*x*_^*e*^) from the values of *p*_*x*_^*e*^.

The actual sex- and age-specific prevalences of ADL and non-ADL limitations were estimated by summing the empirical proportions of (i) persons living at home with restrictions, (ii) inpatients in hospitals and clinics, and (iii) long-term care residents. The counterfactual prevalences after eliminating a given disorder were estimated similarly after first excluding persons with the given disorder.

According to the Sullivan method [7], years of life in age group *x* (*e*_*x*_^*e*^) expected after eliminating a disease are divided into those with or without activity limitation, as follows:

$$ {e}_x^e=\varSigma {\pi}_y^e{L}_y^e/{l}_x^e+\varSigma \left(1-{\pi}_y^e\right){L}_y^e/{l}_x^e $$

Here, Σ represents the sum from age group x to the oldest age group (y ≥ x). π_*y*_^*e*^ is the age-specific prevalence of activity limitation after eliminating the disorder. The years with activity limitation expected after eliminating a disorder were divided into those due to ADL limitations and those due to non-ADL limitations.

## Results

The cause-specific death rate and proportions of persons with and without limitations for each selected disorders by age group in males and females are shown in Tables 1 and 2, respectively. All selected musculoskeletal diseases and injuries were associated with low death rates. Arthrosis and low back pain affected large proportions of outpatients in both the 0 to 64 years and the 65 years or older age groups. Among the selected disorders, fracture led to the frequency of limitations among outpatients.

**Table 1:** Death rate, prevalence of selected diseases and injuries by age group in males

	Death rate (per 100,000 population)	Prevalence (per 1000 population)					
		Residents admitted to facilities^a^	Inpatients^b^	Outpatients^c^			
				Total	No limitation of activities	Non-ADL limitation	ADL limitation
Age 0–64 years							
All diseases and injuries	198.4	0.1	4.1	262.2	211.6	34.5	16.1
Rheumatoid arthritis	0.1	0.0	0.0	1.3	0.7	0.3	0.3
Arthrosis	0.0	0.0	0.0	6.1	3.5	1.7	0.9
Low back pain	0.1	0.0	0.1	23.1	15.9	5.0	2.2
Osteoporosis	0.0	0.0	0.0	0.3	0.2	0.1	0.0
Fracture	2.1	0.0	0.2	3.8	1.8	1.2	0.9
Arthrosis and low back pain	0.1	0.0	0.1	27.8	18.7	6.2	2.8
Age 65 years or older							
All diseases and injuries	3904.6	11.2	27.1	696.1	472.3	124.4	99.4
Rheumatoid arthritis	2.8	0.0	0.0	8.8	4.1	2.4	2.4
Arthrosis	0.1	0.0	0.1	31.2	13.1	9.9	8.2
Low back pain	2.1	0.1	0.3	95.2	43.2	30.7	21.3
Osteoporosis	0.1	0.0	0.0	8.4	2.4	2.3	3.6
Fracture	12.4	0.2	1.2	8.5	2.0	2.3	4.2
Arthrosis and low back pain	2.2	0.2	0.2	116.4	53.2	37.2	26.0

*ADL* Activities of daily living

^a^: Healthcare and welfare facilities for the elderly requiring long-term care

^b^: Inpatients in hospitals and clinics

^c^: Outpatients in hospitals, clinics, and facilities of Japanese traditional massage, acupuncture, moxibustion and judo-orthopedics

**Table 2:** Death rate, prevalence of selected diseases and injuries by age group in females

	Death rate (per 100,000 population)	Prevalence (per 1000 population)					
		Residents admitted to facilities^a^	Inpatients^b^	Outpatients^c^			
				Total	No limitation of activities	Non-ADL limitation	ADL limitation
Age 0–64 years							
All diseases and injuries	102.7	0.1	3.6	284.6	224.7	40.8	19.1
Rheumatoid arthritis	0.1	0.0	0.0	4.9	2.6	1.3	1.0
Arthrosis	0.0	0.0	0.0	11.1	5.4	3.6	2.1
Low back pain	0.0	0.0	0.0	27.1	17.6	6.5	3.0
Osteoporosis	0.0	0.0	0.0	3.9	2.4	0.9	0.6
Fracture	0.7	0.0	0.1	3.1	1.3	0.9	0.9
Arthrosis and low back pain	0.0	0.0	0.1	35.7	22.0	9.2	4.5
Age 65 years or older							
All diseases and injuries	3008.3	30.7	27.9	700.1	442.2	134.1	123.8
Rheumatoid arthritis	5.6	0.2	0.1	19.2	7.6	5.1	6.5
Arthrosis	0.3	0.4	0.5	66.6	26.5	20.5	19.6
Low back pain	1.3	0.4	0.3	126.3	55.1	39.8	31.5
Osteoporosis	0.6	0.3	0.0	95.4	46.4	23.8	25.2
Fracture	13.4	1.3	3.2	20.9	5.1	6.0	9.8
Arthrosis and low back pain	1.5	0.8	0.7	171.5	75.8	52.8	42.9

*ADL* Activities of daily living

^a^: Healthcare and welfare facilities for the elderly requiring long-term care

^b^: Inpatients in hospitals and clinics

^c^: Outpatients in hospitals, clinics, and facilities of Japanese traditional massage, acupuncture, moxibustion and judo-orthopedics

Changes in life expectancy at birth, with and without activity limitation, after eliminating the selected disorders, are shown in Table 3. Life expectancy at birth was 81.0 years in males and 87.1 years in females. The expected years without and with activity limitation were 71.4 and 9.6 years in males and 73.7 and 13.5 years in females, respectively. There were small gains in life expectancy from elimination of rheumatoid arthritis, arthrosis, low back pain, osteoporosis, fracture, arthrosis, and low back pain (0.0–0.1 years). Elimination of rheumatoid arthritis, osteoporosis, and fracture slightly increased the expected years without activity limitation (0.1–0.4) and slightly decreased years with activity limitation (0.1–0.4 years). Elimination of arthrosis, low back pain, and arthrosis and low back pain moderately increased expected years without activity limitation (0.3–1.5 years) and decreased years with activity limitation (0.3–1.5 years).

**Table 3:** Baseline and gains in years with and without activity limitation, at birth, expected from the elimination of selected diseases and injuries

		Life expectancy at birth	Expected years at birth			
			Without activity limitation	With activity limitation	With non-ADL limitation	With ADL limitation
Males	At baseline	80.98	71.36	9.62	5.16	4.46
	Gains from the elimination of					
	rheumatoid arthritis	0.01	0.06	−0.06	−0.03	−0.03
	arthrosis	0.00	0.27	−0.27	− 0.17	− 0.10
	low back pain	0.00	0.75	− 0.75	− 0.54	− 0.20
	osteoporosis	0.00	0.06	−0.06	− 0.02	− 0.04
	fracture	0.07	0.26	−0.20	−0.07	− 0.13
	arthrosis and low back pain	0.00	0.94	−0.94	−0.68	− 0.26
Females	At baseline	87.14	73.65	13.48	6.54	6.94
	Gains from the elimination of					
	rheumatoid arthritis	0.02	0.22	−0.21	−0.10	−0.11
	arthrosis	0.00	0.63	−0.63	−0.40	− 0.23
	low back pain	0.00	1.06	−1.05	−0.80	−0.25
	osteoporosis	0.00	0.37	−0.37	−0.27	− 0.10
	fracture	0.05	0.36	−0.31	−0.08	− 0.23
	arthrosis and low back pain	0.00	1.52	−1.52	−1.13	−0.39

*ADL* Activities of daily living

At birth, the expected years with non-ADL and ADL limitations were 5.2 and 4.5 years in men and 6.5 and 6.9 years in women, respectively. Elimination of rheumatoid arthritis, arthrosis, low back pain, osteoporosis, and fracture decreased expected years with ADL limitations (0.0–0.8 years) and non-ADL limitations (0.0–0.3 years). A combination of arthrosis and low back pain showed a moderate decrease in expected years with both ADL limitations (0.7–1.1 years) and non-ADL limitations (0.3–0.4).

## Discussion

The present study examined potential gains in healthy life expectancy following elimination of musculoskeletal diseases and injuries, using recent national health statistics data in Japan. Our results indicate that musculoskeletal diseases and injuries generally decreased expected years from birth with activity limitations. In particular, low back pain and/or arthrosis decreased expected years from birth with activity limitations to the greatest extent. These findings provide clinical evidence that among the selected musculoskeletal disorders considered here eliminating low back pain and arthrosis are the key factors for the elongation of healthy life expectancy.

A plethora of clinical research indicates that arthrosis of the knee and hip, and low back pain significantly affect medical, economic, and social status. An interesting study showed that on average, patients with chronic low back pain would accept a 7 to 10% reduction in overall life expectancy to ensure that their remaining years were spent pain-free [23]. A clinical study reported that the personal and societal impact of low back pain is very high in patients who have sought multidisciplinary spine care [24]. In particular, the quality of life and workability are low and health care costs are twice as high as those of patients seeking primary low back pain care [24]. Another study showed a reduction in the working life expectancy (i.e., average number of years in employment) of patients with arthrosis and low back pain in the Canadian population [25] and that these patients mainly independently managed the associated limitations these conditions can cause [13, 25]. In the present study, recent Japanese health statistics data showed that arthrosis and low back pain were associated with low death rates and affected large proportions of outpatients in all age groups. In addition, we observed that eliminating low back pain and/or arthrosis decreased expected years from birth with activity limitation to the greatest extent in context of selected diseases and injuries. Consistent with our findings, odds ratios and population attributable fractions associated with various diseases/injuries with activity limitations indicated that orthopedic diseases significantly affect activity limitation [15].

The Japanese statistics showed that the elderly population is increasing, and this trend is expected to continue in the future [26]. Therefore, extended disability-free life expectancy is the only hope of the general population. In this context, increasing public awareness and gaining knowledge of the condition and management strategies of healthy life expectancy is essential. Considering that the “elimination” process performed in our analysis is a hypothetical method, our data do not directly support the promotion of medical management or treatment for these musculoskeletal disorders. However, in line with the above studies, our present study provides clear evidence that musculoskeletal diseases and injuries affect healthy life expectancy, and can have medical, economic, and social implications.

This study has several limitations. First, although there are various musculoskeletal diseases/injuries and their classification, this study examined only five musculoskeletal diseases/injuries as we were limited to diseases that were included in the Comprehensive Survey of Living Conditions. However, the five selected disorders are among the most important musculoskeletal diseases/injuries. Second, ICD-10 codes were assigned to each disease according to the disease classification of the Patient Surveys in the present study, but it is difficult to assign them identically. Third, the disease information in the Patient Surveys and the Survey of Institutions and Establishments for Long-term Care were based on the diagnosis of the doctor and the nursing specialists, respectively. However, because the disease information in the Comprehensive Survey of Living Conditions was self-reported by the patient, the accuracy may have been limited. Fourth, the underlying causes of death used in national health statistics do not account for the indirect effects of the disease on mortality. In addition, the primary disease in national health statistics does not take into account the effect of secondary diseases. Therefore, the impact of musculoskeletal diseases/injuries on the overall life expectancy and healthy life expectancy in this study could have been underestimated and be smaller than it really is. Lastly, the calculation method used in the present study has been adopted in previous studies [14], but it is based on the assumption that “the age-specific prevalence of disability in the stationary population is equivalent to that observed in the real population.” Nevertheless, this study clearly demonstrated that elimination of musculoskeletal diseases and injuries decreased the expected years from birth with activity limitation.

## Conclusions

In conclusion, the present study provides evidence that gains in years with and without activity limitation are expected from eliminating selected musculoskeletal disorders in Japan. Our results suggest that the elimination of low back pain and/or arthrosis would moderately increase healthy life expectancy. These findings provide clinical evidence that among the musculoskeletal disorders low back pain and arthrosis are the key factors for the elongation of healthy life expectancy.

## Data Availability

The datasets used and/or analysed during the current study are available from the corresponding author on reasonable request.

## Abbreviations

ADL: Activities of daily living
WHO: World health organization
